# Depression in Patients with Mastocytosis: Prevalence, Features and Effects of Masitinib Therapy

**DOI:** 10.1371/journal.pone.0026375

**Published:** 2011-10-21

**Authors:** Daniela Silva Moura, Serge Sultan, Sophie Georgin-Lavialle, Nathalie Pillet, François Montestruc, Paul Gineste, Stéphane Barete, Gandhi Damaj, Alain Moussy, Olivier Lortholary, Olivier Hermine

**Affiliations:** 1 Université Paris Descartes, Sorbonne, Paris Cité, Service d'hématologie, Centre de référence des mastocytoses, Hôpital Necker Enfants malades, Paris, France; 2 Université Paris Descartes, Sorbonne, Paris Cité, Laboratoire de Psychopathologie et Processus de Santé EA 4057, IUPDP Institut de Psychologie, Paris, France; 3 Université de Montréal, Centre de Recherche du CHU Sainte-Justine, Montréal, Canada; 4 CNRS UMR 8147, Université Paris Descartes, Sorbonne, Paris Cité, Hôpital Necker-Enfants malades, Paris, France; 5 AB Science, S.A., Paris, France; 6 Département de dermatologie, Centre de référence des mastocytoses, Hôpital Tenon, Université Pierre et Marie Curie, Paris, France; 7 Service d'hématologie, CHU d'Amiens, Université Jules–Vernes Picardie, Amiens, France; 8 Association Française pour les initiatives et la recherche sur les mastocytes et les mastocytoses (AFIRMM), Paris, France; 9 Université Paris Descartes, Sorbonne, Paris Cité, Service de maladies infectieuses et tropicales, Centre de référence des mastocytoses, Hôpital Necker Enfants malades, Paris, France; Chiba University Center for Forensic Mental Health, Japan

## Abstract

Depression in patients with mastocytosis is often reported but its prevalence and characteristics are not precisely described. In addition, the impact of therapies targeting mast cells proliferation, differentiation and degranulation on psychic symptoms of depression have never been investigated. Our objective was to determine the prevalence and to describe features of depression in a large cohort of mastocytosis patients (n = 288) and to investigate the therapeutic impact of the protein kinase inhibitor masitinib in depression symptoms. The description of depression was based on the analysis of a database with Hamilton scores using Principal Component Analysis (PCA). Efficacy of masitinib therapy was evaluated using non parametric Wilcoxon test for paired data within a three months period (n = 35). Our results show that patients with indolent mastocytosis present an elevated prevalence of depression (64%). Depression was moderate in 56% but severe in 8% of cases. Core symptoms (such as psychic anxiety, depressed mood, work and interests) characterized depression in mastocytosis patients. Masitinib therapy was associated with significant improvement (67% of the cases) of overall depression, with 75% of recovery cases. Global Quality of Life slightly improved after masitinib therapy and did not predicted depression improvement. In conclusion, depression is very frequent in mastocytosis patients and masitinib therapy is associated with the reduction its psychic experiences. We conclude that depression in mastocytosis may originate from processes related to mast cells activation. Masitinib could therefore be a useful treatment for mastocytosis patients with depression and anxiety symptoms.

## Introduction

Mastocytosis is a rare disease characterized by mast cells (MC) accumulation in one or several organs [1]–[3]. Based on organ dysfunction, systemic mastocytosis is divided into indolent and aggressive disease [1], [4], [5]. In the majority of cases (>90%) mastocytosis presents as an indolent disease [6]. Even though mastocytosis is usually not a life threatening disease, indolent forms are associated with significant disability in more than 60% of patients [7]. As such, it can have a significant negative impact on quality of life.

Stem cell factor (SCF) is a growth factor that stimulates the proliferation, the survival and the development of mast cells [8]. The biological activity of SCF is induced following its binding to its receptor (SCF-R) encoded by the c-kit proto-oncogene. SCF-R/KIT (also called CD117) is a member of the type III receptor protein-tyrosine kinase family (TKR) [9]. Type III TKRs consist of a glycosylated extra-cellular ligand-binding domain followed by a transmembrane and a cytoplasmic domain which provides docking sites for adaptor proteins following receptor activation allowing cell signalling [10]. Gain of function mutations in c-kit result in constitutive KIT activation and are related to mastocytosis but also other neoplastic transformations including leukaemias, melanoma and others types of cancer [11], [12]. These mutations induce constitutive receptor autophosphorylation and its ligand-independent activity [13]. In most of adult forms of indolent systemic mastocytosis, KIT is constitutively activated as a consequence of D816V mutation, whereas in pediatric forms of mastocytosis extracellular and juxtamembrane mutations are more common [14], [15].

Treatment of mastocytosis is mainly symptomatic, except in aggressive and systemic forms with severe disabling symptoms. Masitinib is a phenylaminothiazole-type tyrosine kinase inhibitor that selectively targets KIT, platelet-derived growth factor receptor (PDGFR) and Lyn kinase [16]. Masitinib's inhibitory effect results in cell cycle arrest and apoptosis of cell lines dependent on KIT signaling [17]. In addition, masitinib inhibits constitutive activation of KIT as a result of juxtamembrane and extracellular mutations found in pediatric forms of mastocytosis. In contrast, activation of KIT as a consequence of D816V mutation is not inhibited by masitinib. However, by blocking Lyn masitinib may block mast cell degranulation and therefore improve symptoms related to mast cell degranulation. Thus, masitinib may have a cytotoxic effect and may reduce tumor burden in patients bearing pediatric's type mutations or wild type c-kit [16].

Although some reported cases of systemic mastocytosis with neurologic manifestations (neurosensory deafness, loss of consciousness, encephalopathy, hypoxic lesions leading to Parkinsonism), suggest central nervous system involvement, it is unsure whether it can be attributed to mast cell infiltration and/or mediators releasing [18]–[21]. Masitinib is a tyrosine kinase inhibitor (TKI) with exhibiting high affinity and selectivity *in-vitro* for KIT receptor and efficiency *in vivo*, because this molecule has been successfully tested in symptomatic patients with systemic mastocytosis [16], [22]–[24]. A phase 2a, multicenter open-label trial over 12 weeks using masitinib showed a strong impact reducing depression by 43% as compared with baseline [24]. This unexpected effect conveyed physician's attention to the links between physiopathological mechanisms inhibited by masitinib and depression in mastocytosis. Masitinib decreases mast cells burden and tryptase levels and may influence depression through mechanisms associated to one of these aspects.

Patients with mastocytosis present many psychopathological manifestations. These manifestations are mainly characterized by cognitive impairment (low attention span, difficulty concentrating and forgetfulness) and negative emotionality (depression, poor motivation, susceptibility to stress, irritability and anxiety). Depression seems to be the most common (sometimes the major) complaint among patients with mastocytosis and it has a profound impact in current professional, social and emotional life. Prevalence rates of depression among these patients range from 40% to 70% [7], [25], whereas prevalence rate is around 7% in the general population and 10%–50% in clinical samples with advanced chronic condition such as cancer or 14%–25% in advanced diabetes [26]–[30]. In addition, recent research in mastocytosis has found dissociation between the objective illness physical impact and depression suggesting that core aspects of depression in this condition are not secondary to the consequence of physical involvement but more a primitive feature of the disease [7].

One of the instruments commonly used to identify depression in patients in clinical trials (including those with mastocytosis) is the 17 items Hamilton Depression Scale (Ham-D17). However, the difficulty in using Ham-D17 to assess depression in mastocytosis may reside in the fact that patients frequently present a vast number of somatic symptoms including insomnia, muscular pain, headache, flush or gastro-intestinal disorders which could in some cases match with Ham-D17 items. Thus, mastocytosis patients presenting a large amount of somatic manifestations could have a high score in Ham-D17 without actually presenting core symptoms of depression such as depressive mood, psychic anxiety, psychomotor retardation or guilt. This means that when exploring depression and treatment effects in mastocytosis we first need to consider the core components of depressive symptoms. That is the reason why we first wanted to describe depression, its structure and symptoms in a large cohort of patients with mastocytosis. Secondarily, we intended to explore changes in depression components and symptoms following a masitinib treatment used to reduce clinical symptoms of mastocytosis.

## Results

### Features and prevalence of depression in mastocytosis patients (N = 288)

**Figure 1:**
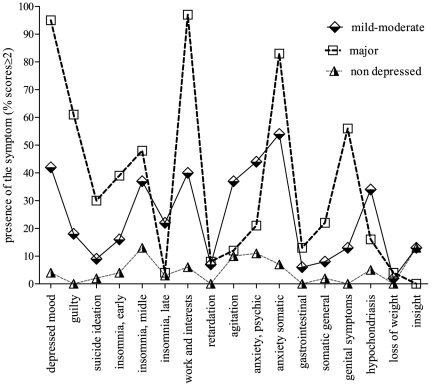
Symptoms of depression from the Ham-D17 in non depressed, mild-moderate and severe depressed patients with mastocytosis. Chi square test was performed to compare groups item by item. Severe depression was characterized by a huge prevalence of impairment in work and activities (97%), depressed mood (95%), somatic anxiety (83%), guilt (61%) and genital symptoms (56%). Mild-moderate depression was characterized by a higher prevalence of late insomnia (22%), agitation (37%), psychic anxiety (44%) and hypochondriasis (34%).

The prevalence rate of mild-moderate depression (Ham-D17 scores ≥ 8 and ≤22) in our sample (67% females; mean age = 47; standard deviation (S.D.)  = 13.58) was 56% and severe depression (Ham-D17 scores ≥23) was observed in 8% of cases (n = 23). In order to know which Ham-D symptoms were mostly prevalent in the sample we analyzed the frequency of patients reporting scores ≥ 2 according to the severity of depression (Fig. 1). Patients presenting severe depression differed significantly from those presenting mild-moderate depression in all items scored ≥ 2 (p≤0.005) except the following: middle insomnia which was very prevalent in both groups (37%–48% respectively), psychomotor retardation, gastrointestinal symptoms and loss of weight which were rare in both groups (2%–3%). Severe depression was characterized by a very high prevalence of impairment in work and activities (97%), depressed mood (95%), somatic anxiety (83%) and guilt (61%). Genital symptoms (56%), early insomnia (39%) and suicide ideation (30%) were also very regular. General somatic symptoms (22%), psychic anxiety (21%) and hypochondriasis (16%) were less common in this group. Others symptoms were rare or only slightly present (less than 13%). When comparing these groups, patients with mild-moderate depression presented significantly (p≤0.0005) more late insomnia (22%), agitation (37%), psychic anxiety (44%) and hypochondriasis (34%).

### Depression in mastocytosis is composed by psychic depression symptoms and sleep disturbances

The Ham-D17 items of the final sample (N = 288) were submitted to a first Principal Component Analyses (PCA) and a promax rotation with a power of 4. Two stable components were found explaining 36.4% of the overall variance (27.96% and 8.38% respectively). Items with loadings under 0.40 were considered lacking sufficient explanation and were excluded from the dimensions. These two components were moderately correlated (r = 0.48). Based on this first PCA and excluding for items suicide and insight which had insufficient loadings, a two-factor solution appeared with a first dimension (items: depressed mood, feelings of guilt, work and activities impairment, psychomotor retardation, agitation, psychic anxiety, somatic anxiety, general somatic symptoms, genital symptoms and hypochondriasis) including most of the core symptoms of depression along with a dimension representing sleep and gastrointestinal disturbances (items: early, middle and late insomnia, gastrointestinal symptoms and loss of weight). To ascertain the consistency of these dimensions, we analyzed inter-item correlations and item-total correlations in each. In the first dimension most inter-item correlations ranged between 0.41 and 0.53. In the second dimension, only middle and late insomnia were correlated with correlations superior to 0.40 (r = 0.45; p<0.001). The following items had correlations under 0.40: early insomnia, psychomotor retardation, agitation, genital and gastrointestinal symptoms, hypochondriasis, loss of weight and loss of insight.

Based on the study of these correlations, we performed a second PCA with a promax rotation and a power of 4 including only the items with inter-item correlations exceeding 0.40 (depressed mood, guilt, middle and late insomnia, psychic and somatic anxiety, work and interests impairment and general somatic symptoms). This PCA resulted in a two components solution explaining 54.55% (41.4% and 13.15% respectively) of the overall variance and moderately correlated (r = 0.42). The first dimension included the psychic symptoms of depression: depressed mood, guilt, work and activities impairment and anxiety (psychic and somatic). We called this dimension “Anxious-depression”. The second dimension included the items middle and late insomnia and was called “Sleep disturbances” (Table 1). The correlation between this version of the scale and the 17 items version was 0.95 (p<0.001). Based on these results further investigations were done on these two components in addition to the traditional Ham-D17 items and score.

**Table 1:** Factor loadings after promax rotation.

Hamilton depression items	Dimensions	
	anxious-depression	Sleep disturbances
depressed mood	0.755	0.121
Guilt	0.657	0.151
work and activities	0.677	0.248
psychic anxiety	0.681	0.235
somatic anxiety	0.686	0.070
general somatic symptoms	0.610	0.121
middle insomnia	0.191	0.819
late insomnia	0.158	0.832

Two components solution explaining 54% of the overall variance and moderately correlated (r = .46) after exclusion of items with correlation loadings under .40. “Anxious-depression” dimension included the following items: depressed mood, guilt, work and activities and anxiety (psychic and somatic). “Sleep disturbances” dimension included the items middle and late insomnia. The Keiser-Meyer-Olkin measure of sampling adequacy revealed a score of 0.85 confirming the adequacy of the data for factor analysis.

### Depression improved following masitinib therapy

**Figure 2:**
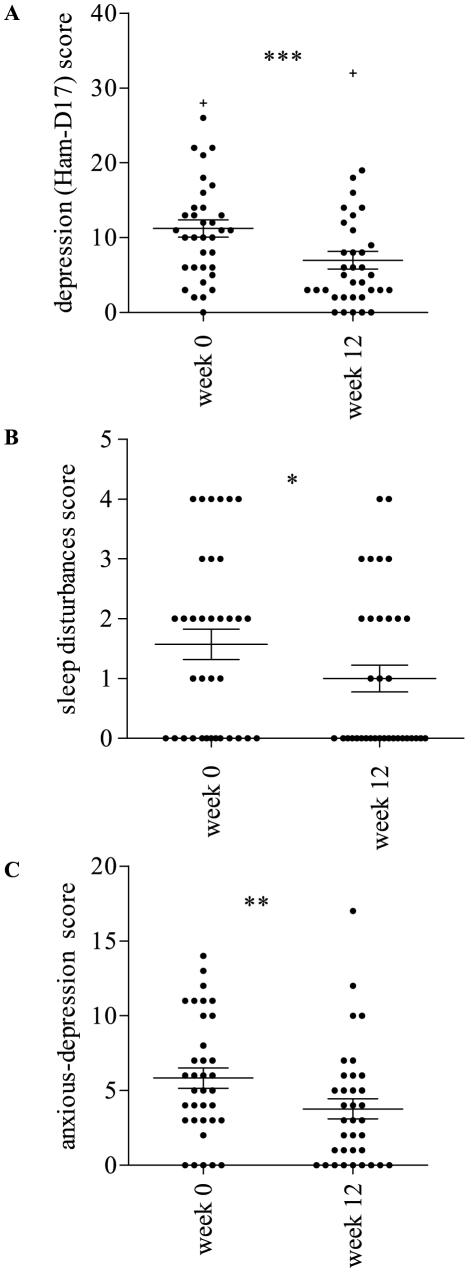
Depression improvement following masitinib therapy. A. Mean total score in depression reduced significantly at the end of the trial (p = .0001); pre-mean Ham-D17 = 11.23 (S.D. = 6.8); post-mean Ham-D17 = 6.97 (S.D. = 6.9). B. Mean score in sleep disturbances dimension reduced significantly at the end of the trial (p = .0112); pre-mean Ham-D17 = 1.57 (S.D. = 1.5); post-mean Ham-D17 = 1.00 (S.D. = 1.3). C. Mean score in anxious depression dimension reduced significantly at the end of the trial (p = .0004); pre-mean Ham-D17 = 5.83 (S.D. = 4.0); post-mean Ham-D17 = 3.77 (S.D. = 3.9). + Exclusion of this patient from the analysis did not change the results. * (p≤0.05); ** (p≤0.0005); *** (p≤0.0001).

At the beginning of the trial (week 0) among 35 patients, 69% presented mild-moderate (n = 22, 92%) or severe (n = 2, 8%) depression. At the end (week 12), only one initially non depressed patient presented a mild depression score (Ham-D17 = 14) and 8 (33%) of the baseline depressed patients did not present any improvement (Table S1). Depression improvement was considered as a reduction of at least 20% of the initial score [31]. Changes in depression scores were significant (p = 0.0001, d = 0.63) and depression improvement concerned 67% (n = 16) of the cases (Fig. 2A). Among improved patients, 75% (n = 12) presented remission as defined by a score ≤7 [32]. Mean scores in anxious depression and sleep disturbances dimensions were also significantly reduced after masitinib therapy (p = 0.0004, d = 0.52 and p = 0.0112, d = 0.41 respectively) (Fig. 2B/C). In order to provide data comparable with pharmacological studies analyzing classical depression treatment response as with fluoxetine we tested response rate using similar depression baseline cut-point of Ham-D17≥16 and a decrease of at least 50% in final score to consider response to masitinib. Using these criteria, 8 patients (23%) were included in the depression group. The response rate was 50% and 25% displayed a remission score of HAM-D ≤7 (2 patients) (Table S1).

### Depression improvement after masitinib therapy was not related to improvements in global quality of life

In order to know whether improvement of depression after masitinib therapy could be attributed to global quality of life (gQoL) improvement we investigated the relations between these evolutions. Global quality of life levels (mean week0  = 47.20; S.D.  = 21.64, mean week12 = 58.35; S.D.  = 20.73) slightly improved after mastinib therapy (p = 0.043, d = 0.53). Improvement in gQoL (defined as an increase of at least 20% in score) concerned 43% (n = 15) of patients; 17% (n = 6) were worsened and 40% (n = 14) did not display any changes. Among improved patients, only 4 presented an increase of at least 50% in gQoL scores. Baseline gQoL scores were negatively correlated to baseline scores in depression showing that a better global quality of life was related to lower depression scores at baseline (r =  −0.471; p = 0.004). At the end of the trial, global gQoL was not correlated to depression scores (total and dimensions) (r = 0.077 to −0.287; ns). We computed relative change indices by dividing absolute raw changes by baseline raw values. Relative changes in gQoL were unrelated to relative changes in depression scores (r = −0.041; p = 0.815).

## Discussion

In the present study we found in a sample of 288 patients with mastocytosis a high prevalence of depression (64%) and a significant improvement of depression following masitinib therapy.

Depression is a frequent comorbidity among patients with physical illness and it is associated to increased disability [33]. In this work we showed that patients with systemic indolent mastocytosis present an elevated prevalence of depression (64%) confirming and extending results from previous studies [7], [25]. However, this study was the first to evaluate the severity of depression in these patients. Our results show that depression in mastocytosis is mainly (56% of the cases) moderate whereas severe depression concerned 8% of depressed patients. This high prevalence is surprising because indolent forms of mastocytosis are not life threatening as is cancer and yet, depression prevalence rates are comparable or even higher than in this latter condition [26]–[28], [30].

We showed that depression among mastocytosis patients was predominantly characterized by anxious and depressive symptoms. Physical symptoms as gastrointestinal complaints, general symptoms and loss of weight were only slightly present in mastocytosis depressed patients. This suggests that depression among patients presenting indolent forms of the disease is rarely accompanied by appetite loss or anorexia. Consequently, ‘loss of weight’ was rarely scored. More surprising was the low prevalence of general somatic symptoms as they reflect pain (cephalic, muscular or articular) suggesting that these symptoms are often related to mastocytosis but not as much to depression among these patients. Psychomotor retardation and loss of insight were rare and should be considered as atypical symptoms in this population. Interestingly, hypochondriasis was more prevalent among mild-moderate depressed patients while genital complaints were more prevalent among severe depressed patients. This result suggests that severe depressed patients with mastocytosis are less concerned or at least less apprehensive about their bodily sensations and health state than mild-moderate depressed patients but are more impaired in sexuality.

Only 8% of patients presented a severe depression score. However, as might be expected, these severely depressed patients displayed a high frequency (30%–95%) of guilt, suicidal ideation and depressed mood. The high frequency of this group of symptoms is a warning for a risk of suicidal attempt that should be more seriously taken into account in severe depressed patients with mastocytosis as it is in people with other conditions. Our results suggest that depression in mastocytosis has a specific pattern different from those in other physical conditions. For example, in advanced diabetes, depression is characterized by cognitive/affective symptoms [34]. These very same symptoms were also associated with cardiovascular prognosis in patients with post-myocardial infarction [35]. In mastocytosis, depression does not seem to be related to physical related symptoms or severity and therefore should be considered as an endogenous manifestation.

In this study, we evaluated the response to masitinib without placebo condition among a cohort from an open-label trial. Indeed, masitinib is a tyrosine kinase inhibitor with a specific action on MC [16], [22]–[24]. Masitinib has already showed efficacy in the treatment of cutaneous mastocytosis [24]. Although in this study a placebo control was absent, our results showed impressive response rates for masitinib therapy on depression associated with mastocytosis. Masitinib treatment was associated with significant improvement in depression (67% of responders and 75% of remitted patients among responders). We also used different criteria for depression (Ham-D17≥16) and response (a decrease of at least 50% of scores in Ham-D17) and found a response rate of 50% and a remission rate of 25%. As a comparison, fluoxetine, a widely used antidepressant drug, has been associated with a response rate of 60% and a remission rate of 58% after 10 weeks of treatment in a sample of patients without mastocytosis, but with comparable baseline depression levels and same criteria for response [36]. These results bring evidence to anti-depressant effects of masitinib in the context of mastocytosis. However, more research is needed to confirm masitinib efficacy on depression using controlled trials and larger samples.

Quality of life in mastocytosis is often impaired because of the chronicity of some symptoms but most patients do not experience severe functional disabling. In our previous work we have shown that the impact of mastocytosis symptoms in quality of life was related to the patients' subjective perception of their own symptoms [7]. The masitinib efficacy in bringing relief to physical and clinical symptoms of mastocytosis has been demonstrated [24]. It has been shown in cancer or diabetic patients that poor quality of life is related to symptoms of depression and anxiety [29]. Therefore, we wanted to explore effects of masitinib in depression and if they were independent of gQoL. We showed that despite a relation between a deteriorated quality of life and depression at baseline, improvements in gQoL did not explain improvements in depression after masitinib therapy. This last result suggests that depression in mastocytosis does not improves in relation to a better QoL. This result is in line with our hypothesis that depression in mastocytosis represents a systemic neurological manifestation of the disease besides other physical symptoms. Instead, it suggests that improvements in depression could be influenced by the inhibitory effect of masitinib on mast cells activation and cerebral dysfunction underlining the systemic nature of depression in this condition. This strongly suggest that depression in mastocytosis is a primary systemic symptom of the disease rather than only a secondary psychological distress reaction and that masitinib could reduce neuropsychological symptoms by specifically targeting MC degranulation and migration.

We suggest that the configuration of depression in mastocytosis could be articulated into two dimensions: *i.* an “anxious-depression dimension” including the symptoms reflecting core cognitive, affective and somatic aspects of depression (depressed mood, guilt, work and interests impairments) and anxiety (somatic and psychic anxiety), and *ii.* a “sleep disturbances dimension” grouping sleep disturbances related to depression (middle and late insomnia). When exploring depression in mastocytosis, symptoms usually considered as the core of depression (psychic anxiety, depressed mood, work and interests) characterized depressed patients. In a subsample of 35 patients, Masitinib therapy was associated with significant improvement (50%–67% of the cases) of overall depression, with 25%–75% of recovery cases according to criteria for baseline depression and improvement. Response in depression symptoms after masitinib therapy was demonstrated through improvement of the anxious-depression dimension as well as of total scores. Global quality of life slightly improved after masitinib therapy but these changes did not predict depression improvement. These results suggest a specific MC involvement in psychological symptoms present in this disease.

This huge prevalence of depression suggests a systemic brain involvement probably through MC mediators such as serotonin, substance P or cytokines. Recent research suggested that mast cells are involved in mechanisms related to emotion regulation [37]–[39]. Mast cells are often located around vessels and it has been shown that, even if mast cells do not cross the blood-brain-barrier (BBB), these cells can be implicated in inflammatory process through mediators releasing that will increase permeability of the BBB [40]–[44]. Indeed, MC are involved in many other diseases including neuroinflammatory conditions such as irritable bowel syndrome, multiple sclerosis, asthma, atopic dermatitis, fibromyalgia, migraine, rheumatoid arthritis, scleroderma, etc [45]. In these conditions, negative emotionality such as anxiety is also common among patients [37]. This relation between MC and negative emotions (i.e. anxiety) has also been demonstrated in rodents suggesting a conservation of this relation through evolution and an involvement of MC in aspects of emotionality [39]. The presence of MC in brain regions; at the proximity of nerves and vessels, could sign a primary MC involvement in neurobiological determinants of depression [39], [46]–[48]. In the brain, many MC reside also in the thalamus [49]–[55]. Lesions or stimulation of the thalamus (medial dorsal and anterior nuclei) are associated with changes in emotional reactivity [53]–[55]. In addition, the regulation of emotional behavior is due to the connections of the nuclei of thalamus with other limbic system structures [56], [57]. We could hypothesize that masitinib could reduce depression symptoms in mastocytosis by two pathways: *i.* by blocking mediators secreted by mast cells involved in increasing permeability of the BBB and brain inflammation; *ii*. by reducing mast cells numbers in the thalamus or other structures of the limbic system through its direct effect on KIT and Lyn.

In this study we have analysed data from cohort of patients derived from a multicenter, open-label phase 1a and phase 2a trial. Therefore there was no group of patients with mastocytosis displaying depression which were not treated by masitinib to show that in this case depression scores remains stable between baseline and the end of the study. Secondly, although we used a validated measure of depression, this is not a structured diagnostic interview, which is usually considered as the gold standard to detect clinical depression. Further research should address these limitations and explore the link between MC and psychological manifestations, such as depression and anxiety in mast cell related diseases.

In conclusion, our results suggest that depression in mastocytosis may share common underlying processes related to the disease and/or MC Our results suggest that a pharmacological intervention, specifically targeting MC, alone brought a satisfactory improvement of depression. As it has been evidenced in other conditions such as obesity and diabetes, a systemic origin common to mastocytosis and depression could be searched for. In diabetes for example, a large part of depression has been suggested to be systemic rather than secondary because both conditions have been related to common underlying biological processes including inflammation processes [58], [59]. This may also be applicable to mastocytosis and would explain why we observe such a dramatic decrease of depression following masitinib. Furthermore, brain mast cells have been shown to evoke hypothalamic-pituitary-adrenal responses via centrally released histamine and corticotrophin-releasing factor [51]. Also, the hypersecretion of cortisol and the dysfunction of the hypothalamo-pituitary-adrenal axis seem to be implicated in medical co-morbidities associated with mood disorder [60]. The results underline the fact that masitinib not only affect mastocytosis physical symptoms as it has been shown [24], but also that it seem to favourably impact depression and anxiety symptoms, by targeting specifically MC.

## Methods

### Sample description (Table 2)

We analyzed a database containing Ham-D17 scores for 288 patients identified by the Association Française pour les Initiatives et Recherche sur les Mastocytes et la Mastocytose (AFIRMM) and tested between 2003 and 2007. All patients had a proven diagnosis of mastocytosis following the WHO criteria [61]. From this large sample, 35 patients were included in a pharmacotherapeutical phase 1a (n = 25) and phase 2a (n = 25) multicenter, open-label trial to evaluate the response to masitinib in indolent mastocytosis with handicap, with a 3-month interval between the baseline (week 0) and final (week 12) tests [24]. We retained for our study only patients with complete measures in week 0 and week 12, thus 15 patients were excluded.

**Table 2:** Sample description.

*Afirmm protocol (N = 288)*	
Gender	98 men/163 women
Age in years (S.D.) – (range)	47 (13.58) – (20–82)
Ham-D17 total score (S.D.) – (range)	11.4 (7.7) – (0–35)

*Masitinib trial (N = 35)*	
Gender	11 men/24 women
Age in years (S.D.) – (range)	49 (12.74) – (19–77)
Ham-D17 total score week 0 (S.D.) – (range)	11.23 (6.89) – (0–28)
Ham-D17 total score week 12 (S.D.) – (range)	6.97 (6.90) – (0–32)

S.D: standard deviation; Ham-D17: Hamilton depression scale-17 items.

### Measures

In the whole sample (N = 288), the following measures were available: age, gender, Hamilton scores (Table 2). With the exception of age, missing data represented less than 10% in this sample and an imputation was not necessary. In the follow-up study (masitinib protocols), additional measures of quality of life were taken (measured with the cancer quality of life questionnaire (EORTC-QLQ-C30 version 3).

#### The Hamilton Depression Scale

The Ham-D17 has been mainly used to assess depression in clinical trials and remains a reference measure to evaluate depression in research concerning somatic patients [62]. Ham-D17 is composed of 17 items scored 0-4 (depressed mood, guilt, suicide, psychic and somatic anxiety, psychomotor retardation, agitation, hypochondriasis, work and interests impairment) or 0–2 (early, middle and late insomnia, gastrointestinal, somatic general, genital, loss of weight and loss of insight items) according to the absence, presence and seriousness of the symptom. These items reflect two groups of symptoms: core symptoms of depression (depressed mood, guilt, work and interests impairment, psychic anxiety, psychomotor retardation and general somatic symptoms) and symptoms reflecting secondary aspects and related to cognitive (hypochondriasis, suicide thoughts, loss of insight, agitation), and somatic disturbances (somatic anxiety, gastrointestinal symptoms, genital symptoms, loss of weight, early, middle and late insomnia) [63], [64]. The total score of Ham-D17 is usually used as criterion to appreciate effect of treatment in clinical trials. However, the primary efficacy criterion should focus on the total score of core symptoms since they reflect the clinical response related to more typical aspects of depression [65]. Although the Ham-D17 was not initially designed as a self-report, Reynolds and Kobak developed a paper and pencil version with 17 items corresponding in contents and scoring to the standard Ham-D17 version and which showed excellent reliability [66].

#### Quality of life questionnaire

The European Organisation for Research and Treatment of Cancer Quality of Life Core Questionnaire (EORTC-QLQC30) is a cancer specific questionnaire, reporting health related quality of life in these patients [67]. It is a 30 item self-report instrument with Likert response scales whereby increasing scores indicate increasing burden for the functional and symptom scales. The questionnaire provides a quality of life “global score” (gQoL)” were an increased score indicates a good level of quality of life in addition to the functional and the symptoms scales.

### Procedures

The study was approved by the local ethical committee of Necker hospital, patients were given informed consents and the study was carried out in compliance with the precepts of the Helsinki protocol. Data were collected through a mail-back procedure. Patients were sent questionnaires and had to mail back filled questionnaires within a week. For the follow-up subsample (protocols), patients were interviewed at baseline and at week 12 by a trained clinician.

### Statistical analysis

SPSS version 17.0 0 (IBM SPSS Inc., Chicago, IL, USA) was used for most analysis. Wilcoxon and Chi-squared tests were performed using GraphPad Prism software version 5.01 (GraphPad Software Inc., San Diego, CA). To describe depression, we explored the structure of the Ham-D17 using Principal Component Analyses (PCA) and Chi-square tests. To explore the effect of therapy on depression symptoms, we compared pre-post scores and items using non parametric Wilcoxon test for paired data. To investigate the effects of quality of life on depression improvement after masitinib, we used correlation and linear regression. All reported p values are two tailed with a significance level of .05.

## Supporting Information

Table S1 **Improvements in depression following masitinib therapy according to depression and improvement criteria used.**
(DOCX)
