# Supplementary material for: Depression in Patients with Mastocytosis: Prevalence, Features and Effects of Masitinib Therapy
Source: PLoS One. 2011 Oct 21;6(10):e26375. doi: 10.1371/journal.pone.0026375 (PMC3198767; doi:10.1371/journal.pone.0026375)
Supplement: Table S1 — Improvements in depression following masitinib therapy according to depression and improvement criteria used. (DOCX) [file pone.0026375.s001.docx]

| **Table S1. Improvements in depression following masitinib therapy according to depression and improvement criteria used** | | | | | | | |
| --- | --- | --- | --- | --- | --- | --- | --- |
|  |  |  | Criteria for depression  (week 0 ≥ 8) | | Criteria for depression  (week 0 ≥ 16) |  |  |
|  | Depression score | | % improvement | | |  |  |
| patients | w0 | w12 | 20% | 50% | 50% | remitted  (w 12< 7) | not improved |
| **1** | 3 | 4 |  |  |  |  |  |
| **2** | 4 | 0 |  |  |  |  |  |
| **3** | 12 | 3 | x | x |  | x |  |
| **4** | 8 | 13 |  |  |  |  | x |
| **5** | 28 | 32 |  |  |  |  | x |
| **6** | 10 | 5 | x |  |  | x |  |
| **7** | 6 | 3 |  |  |  |  |  |
| **8** | 13 | 3 | x | x |  | x |  |
| **9** | 12 | 3 | x | x |  | x |  |
| **10** | 10 | 8 | x |  |  |  |  |
| **11** | 6 | 0 |  |  |  |  |  |
| **12** | 6 | 0 |  |  |  |  |  |
| **13** | 3 | 0 |  |  |  |  |  |
| **14** | 11 | 5 | x | x |  | x |  |
| **15** | 10 | 9 |  |  |  |  | x |
| **16** | 2 | 2 |  |  |  |  |  |
| **17** | 16 | 6 | x | x | x | x |  |
| **18** | 11 | 8 | x |  |  |  |  |
| **19** | 13 | 11 |  |  |  |  | x |
| **20** | 11 | 6 | x |  |  | x |  |
| **21** | 26 | 12 | x | x | x |  |  |
| **22** | 8 | 4 | x | x |  | x |  |
| **23** | 5 | 0 | x | x | x | x |  |
| **24** | 21 | 3 | x | x | x | x |  |
| **25** | 0 | 2 |  |  |  |  |  |
| **26** | 6 | 14 |  |  |  |  |  |
| **27** | 2 | 2 |  |  |  |  |  |
| **28** | 22 | 18 |  |  |  |  | x |
| **29** | 18 | 8 | x | x | x |  |  |
| **30** | 14 | 14 |  |  |  |  | x |
| **31** | 14 | 2 | x | x |  | x |  |
| **32** | 13 | 6 | x | x |  | x |  |
| **33** | 10 | 3 | x | x |  | x |  |
| **34** | 17 | 16 |  |  |  |  | x |
| **35** | 22 | 19 |  |  |  |  | x |
| **Depression scores (Ham-D17) of patients at week 0 and week 12 are reported. Using the same criteria as in fluoxetine trials (Ham-D17 ≥ 16 and a decrease of 50% in final score to consider response), 8 patients (23%) were included in the depression group. The response rate was 50% and 2 patients (25%) displayed a remission score of Ham-D ≤ 7 w: week** | | | | | | | |
